# The Prince of Wales's Hospital Fund

**Published:** 1898-09-17

**Authors:** 


### THE PRINCE OF WALES'S HOSPITAL FUND.

### AN ISSUE OF NEW HOSPITAL STAMPS FOR 1898.

Last year two stamps were issued on behalf of this Fund. They produced a sum of about £35,000, and their object was to give small subscribers a handy and convenient form of receipt. This has become popular; for, in conjunction with the stamp albums and subscription books, they afford evidence of the actual sum each person has given to the hospitals through the Prince of Wales's Fund. In 1897 a large number of the public, estimated at about 600,000, gave their subscriptions by purchasing these stamps.

The 1898 issue consists of four stamps, each of distinct design, with a face value of 1s., 2s. 6d., 5s, and 10s. respectively. The designs for the stamps and the colours in which they are printed were selected by H.R.H. the Prince of Wales. The 1s. stamp is printed in red, the 2s. 6d. stamp in blue, the 5s. stamp in dark green, and the 10s. stamp in green. Each stamp contains a vignette of the figure of Charity, with the words "Prince of Wales's Hospital Fund," and the date 1898 in the top right hand corner. A facsimile of the Prince's signature appears at the bottom of each stamp. The 1s., 5s., and 10s. stamps have the Prince of Wales's feathers introduced, whereas in the 2s. 6d. stamp these are replaced by a fleur-de-lis. The 10s. stamp is intended as a national emblem, the vignette being enclosed by an ornamental border, containing the rose, shamrock, thistle, and leek. The designs are strikingly attractive, and their production reflects the greatest credit on Messrs. De La Rue and Co., who have generously presented the dies to the Prince's Fund free of cost. It is evident that stamp collectors who displayed genuine interest in these stamps last year will be largely interested in the early purchase of specimens of the new stamps for their albums, most of which have specially prepared pages for the insertion of the hospital stamps. This year it has been decided to reduce the number of stamps printed to one-third of the number produced in 1897. This step will no doubt increase the public interest in the 1898 stamps, seeing that the plates will be destroyed and no further stamps printed beyond the strictly limited number referred to. It is hoped that the whole of the 340,000 stamps which constitute the entire issue will be taken up between the present time and the end of the year, so that the proceeds may be made available for distribution amongst the hospitals in December next. Messrs. De La Rue and Co. have done the engravings of the originals by hand, and it has been carried out in the most highly finished style on soft steel by a thoroughly skilled and artistic engraver, who has been occupied upon the work for several months. After the engraving was finished the original plates were hardened. The designs were then taken up under a pressure of about twenty to thirty tons upon soft steel rollers, which in their turn had to be hardened. The rollers were then employed for rolling in under a pressure of twenty to thirty tons the required steel printing plates. These plates served for printing the stamps by the ordinary copper-plate printing process. The distribution of the new stamps has been entrusted to Messrs. Simpkin, Marshall, Hamilton, Kent, and Co. (Limited); they will be ready on September 20th, 1898, and may be obtained through any bookseller, stationer, or newsagent, and of most chemists.

